# Therapeutic Patient Education for Severe Mental Disorders: A Systematic Review

**DOI:** 10.1192/bjo.2024.209

**Published:** 2024-08-01

**Authors:** Hafsa Meraj, Ahmed Waqas

**Affiliations:** 1 Greater Manchester Mental Health Trust, Eccles, United Kingdom; 2 University of Liverpool, Liverpool, United Kingdom

## Abstract

**Aims:**

Therapeutic Patient Education (TPE) aims to help patients self-manage their chronic condition over their lifetime, adapting to their evolving circumstances, as well as changes in their condition and treatment. The National Institute for Clinical and Healthcare Excellence underscores the importance of patient education as a crucial part of early interventions for mental disorders. This systematic review aimed to review TPE programmes in managing psychiatric disorders, considering the diversity in delivering agents, intervention formats, targeted skills, and therapeutic outcomes.

**Methods:**

Comprehensive database searches, including Web of Science, PubMed, and COCHRANE, were conducted from September 2019 to January 2023, yielding 514 unique records, with 33 making it through rigorous evaluation for full-text review. Eleven studies met the inclusion criteria, focusing on various psychiatric disorders such as depression, bipolar disorder, psychosis, and multiple serious mental illnesses. A total of 38 studies were included from our previous review to supplement the current database search.

**Results:**

Among 49 included interventions, 13 were aimed at bipolar disorder, depression (n = 12), multiple serious mental illnesses and comorbidities (n = 11), schizophrenia and psychoses (n = 13). A total of 21 interventions were delivered in groups followed by individual (n = 12), mixed format (n = 14) and electronically (n = 2).

TPE programmes exhibited diversity in delivering agents and intervention formats, with a notable presence of multidisciplinary teams and various professionals. The interventions prioritized coping strategies and disease management techniques, though the extent varied based on the disorder. Examining the different skills imparted during the interventions, the focus predominantly leaned towards the teaching of coping strategies. These encompassed both cognitive and behavioural coping skills, including areas such as self-confidence (n = 37), stress management (n = 39), critical thinking (n = 26), problem-solving (n = 18), goal setting (n = 31), situational awareness (n = 36), and self-care (n = 36), with unspecified coping skills also noted (n = 32).

Effectiveness was heterogeneous across studies; some interventions showed significant benefits in areas such as symptom management, coping, and functional improvement, while others reported no significant outcomes.

**Conclusion:**

The findings underscore the potential of TPE in psychiatric care, revealing its multifaceted nature and varied impact. TPE not only addresses deficits but also leverages patients' existing strengths and capabilities. Despite the reported benefits, a portion of the interventions lacked statistical significance, indicating the necessity for continuous refinement and evaluation.

